# Within-Breath Oscillatory Mechanics in Horses Affected by Severe Equine Asthma in Exacerbation and in Remission of the Disease

**DOI:** 10.3390/ani12010004

**Published:** 2021-12-21

**Authors:** Luca Stucchi, Francesco Ferrucci, Michela Bullone, Raffaele L. Dellacà, Jean Pierre Lavoie

**Affiliations:** 1 Dipartimento di Medicina Veterinaria, Università degli Studi di Milano, 26900 Lodi, Italy; luca.stucchi@unimi.it; 2 Dipartimento di Scienze Veterinarie, Università di Torino, 10095 Grugliasco, Italy; michela.bullone@unito.it; 3 Dipartimento di Elettronica, Informazione e Bioingegneria, Politecnico di Milano, 20133 Milano, Italy; raffaele.dellaca@polimi.it; 4 Département de Sciences Cliniques, Faculté de Médecine Vétérinaire, Université de Montréal, Saint-Hyacinthe, QC J2S 2M2, Canada; jean-pierre.lavoie@umontreal.ca

**Keywords:** equine asthma, impulse oscillometry, airway obstruction, lung function test

## Abstract

**Simple Summary:**

Equine asthma shares similarities with human asthma. The aim of the study was to evaluate whether within breath analysis improved the sensitivity of oscillometry at detecting subclinical airway obstruction in horses with asthma in remission of clinical signs. From this study, we can conclude that the within-breath oscillometry is sensitive in discriminating horses with severe asthma in clinical remission of the disease from control horses. Additionally, oscillometry allowed to identify the increase in expiratory reactance similar to that due to expiratory flow limitation observed in human asthmatic patients with airway obstruction.

**Abstract:**

Oscillometry is a technique that measures the resistance (R) and the reactance (X) of the respiratory system. In humans, analysis of inspiratory and expiratory R and X allows to identify the presence of tidal expiratory flow limitation (EFLt). The aim of this study was to describe inspiratory and expiratory R and X measured by impulse oscillometry system (IOS) in horses with severe asthma (SEA) when in clinical remission (*n* = 7) or in exacerbation (*n* = 7) of the condition. Seven healthy, age-matched control horses were also studied. Data at 3, 5, and 7 Hz with coherence > 0.85 at 3 Hz and >0.9 at 5 and 7 Hz were considered. The mean, inspiratory and expiratory R and X and the difference between inspiratory and expiratory X (ΔX) were calculated at each frequency. The data from the three groups were statistically compared. Results indicated that in horses during exacerbation of severe asthma, X during expiratory phase is more negative than during inspiration, such as in humans in presence of EFLt. The evaluation of X during inspiration is promising in discriminating between horses with SEA in remission and control horses.

## 1. Introduction

Horses can spontaneously develop equine asthma, a non-infectious chronic lower airway disorder of adult horses, which shares several similarities with human asthma [1,2]. Based on the severity and the clinical presentation, the disease is classified as mild-moderate or severe equine asthma. Severe equine asthma (SEA) is characterized by coughing, exercise intolerance, and recurrent episodes of increased respiratory effort at rest, representing the exacerbation of the condition, alternated with periods of remission of the clinical signs [3]. The gold standard for the diagnosis of SEA is the cytological examination of bronchoalveolar lavage fluid (BALf) in horses with compatible clinical signs, that shows the presence of a marked neutrophilic inflammation [4]. Nevertheless, BALf collection is a relatively invasive procedure, that requires the sedation of the patient and the instillation of a large volume of fluid in the lungs of the horse. Moreover, during the remission of the clinical signs, BALf cytology may not allow to discriminate between healthy and affected horses [5]. In humans, the gold standard for the asthma diagnosis is the detection of alterations in pulmonary function testing [6]. In horses affected by SEA, conventional lung mechanics allows to identify the presence of airway obstruction [7,8,9]; however, this technique requires the use of an esophageal balloon and shows a low sensitivity for mild obstruction [10], therefore it is currently performed only in research settings. For this reason, in the last 30 years the attention of the researchers has been focused on oscillometry, a technique currently used in humans for the evaluation of asthmatic patients. As described by Dubois et al. [11], oscillometry allows to measure the mechanical properties of the lung (i.e., resistance, R, and reactance, X), evaluating the response of the respiratory system to external forcing over-imposed to spontaneous breathing. The Impulse Oscillation System (IOS) is a method based on a repetition of impulses generated from a loudspeaker and applied to the respiratory system that allows the determination of R and X across multiple frequencies [12]. Van Erck et al. [13,14,15,16] first reported on the use of IOS in horses with severe asthma, in which they evaluated the frequencies from 5 to 20 Hz. They reported that the results of IOS and conventional lung mechanics were well correlated. Moreover, IOS was more sensitive than standard mechanics during bronchoprovocation tests. Lower airway obstruction was characterized by negative frequency dependence of R, positive frequency dependence of X and negative X values throughout the frequency range. In 2006, Klein and colleagues evaluated the results of IOS at frequencies of 1, 5, and 10 Hz, that were considered the most representative of equine lower airways [17]. For the first time, the within-breath analysis was performed, and inspiratory and expiratory R and X were reported. Additionally, the within-breath analysis of asthmatic horses with subclinical inflammation, showed higher R values and lower X values when compared to those of controls [18]. Recently, other authors reported an association between R measured by IOS and histopathological findings of the airways of SEA horses [19]. To date, there is no report of IOS values of SEA when horses are in remission of the clinical signs.

In humans, the within-breath analysis of X allows to calculate the parameter Delta X (ΔX), defined as the difference between the inspiratory and expiratory reactance at each frequency. It has been shown that this parameter allows the detection of tidal expiratory flow limitation (EFLt) in COPD and asthma. This measure has an important diagnostic value [20,21], but it has not been applied for the evaluation of asthmatic horses. The aim of the present work was therefore to describe the results of within-breath analysis, including ΔX, measured by IOS in horses with severe asthma when they are in the exacerbation and in the remission phases of the disease.

## 2. Materials and Methods

### 2.1. Sample Selection

To perform the study, seven horses with SEA in exacerbation of the clinical signs (4 geldings and 3 mares, age of 11.9 ± 3.4 years), seven horses with SEA in clinical remission (1 gelding and 6 mares, aged 16.4 ± 5.0 years) and seven age matched healthy controls (7 mares, with a mean age of 13.1 ± 3.5 years) were studied. The horses with SEA were selected from a well-characterized population of asthmatic horses of the research herd of the Equine Asthma Laboratory, Faculty of Veterinary Medicine, University of Montréal. Horses in asthma exacerbation were kept in stable and fed hay; horses in remission of the clinical signs were kept at pasture 24 h/day for at least 6 months and fed pelleted hay when needed. Control horses were from the teaching herd of the Faculty of Veterinary Medicine of the University of Montréal and were considered free from respiratory diseases based on history and clinical examination. All horses had been previously trained to IOS measurement. The study was approved by the Animal Care Committee of the Université de Montréal (Protocol Rech-1324) and conducted in compliance with the guidelines of the Canadian Council on Animal Care.

### 2.2. IOS Measurement

Horses were restrained in stock and underwent IOS measurement by Equine IOS MasterScreen (Jaeger, Würzburg, Germany), as previously described [13]. Briefly, the system consisted in a plastic mask adapted to fit on the muzzle of the horse, sealed by a rubber tape. The mask was attached through a tube to a loudspeaker that produced the impulses, and to a pneumotachograph placed directly in front of the face mask. The pressure and flow response of the respiratory system to the impulses superimposed to the animal spontaneous breathing were measured. Prior to each experiment, the system was calibrated by means of a 2-L calibration syringe, forcing known volumes of air through the pneumotachograph. At least three measurements of 30 s each were performed, and the mean value of the three measurements was studied.

The data collected by LabManager (version 4.53, Jaeger, Würzburg, Germany) was then analyzed using Fast-Fourier transformation (FAMOS imc, Meβsysteme, Berlin, Germany). The mean total, inspiratory and expiratory R and X and the corresponding coherence (Co) of the respiratory system at all frequencies of impulses (from 0.1 to 20 Hz) was obtained. For this study, only values at 3, 5, and 7 Hz were studied, as they were the only frequencies with Co considered adequate (Co > 0.85 at 3 Hz and 0.9 at 5 and 7 Hz). Co reflects the quality of the measurement [17]. The ΔX, measured as the difference between the mean inspiratory and expiratory reactance at each frequency, was also calculated.

### 2.3. Statistical Analysis

The mean inspiratory and expiratory R and X and the ΔX were calculated at each frequency for the three groups and collected on an electronic spreadsheet (Microsoft Excel, Redmont, WA, USA). Data distribution was evaluated by means of Shapiro–Wilk normality test. If data were normally distributed, the comparison between the three groups was performed by means of one-way ANOVA and Dunnett’s multiple comparison test. If data were not normally distributed, the comparison was performed by Kruskal–Wallis test and Dunn’s multiple comparison test. Statistical analysis was performed using a statistical software (Prism Graphpad 9.1.0 for MacOs; San Diego, CA, USA). Statistical significance was set at *p* < 0.05.

## 3. Results

The Shapiro–Wilk normality test showed a normal distribution for all the parameters, except for inspiratory R at 3 Hz (R3i) and ΔX at 7 Hz (ΔX7). Results of IOS measurement are reported in Table 1.

Statistical comparison between groups showed significant differences between SEA horses in exacerbation and control horses for R at 3 Hz, for mean (R3, *p* = 0.0002), inspiratory (R3i, *p* = 0.0011) and expiratory (R3e, *p* = 0.0008) parameters.

For X, significant differences were present between horses in exacerbation and control horses at each frequency for mean (X3, *p* < 0.0001; X5, *p* < 0.0001; X7, *p* < 0.0001), inspiratory (X3i, *p* < 0.0001; X5i, *p* < 0.0001; X7i *p* = 0.0007), and expiratory parameters (X3e, *p* < 0.0001; X5e, *p* < 0.0001; X7e, *p* < 0.0001). Between control horses and asthmatic horses in remission, differences were present for mean X at 7 Hz (X7, *p* = 0.0173) and for inspiratory X at 3, 5 and 7 Hz (X3i, *p* = 0.009; X5i, *p* = 0.0017; X7i, *p* = 0.012).

The ΔX values were significantly higher in horses in exacerbation of severe asthma than in control horses at 3 and 5 Hz (ΔX3, *p* = 0.0029; ΔX5, *p* = 0.001), indicating a worsening of the airway obstruction during the expiratory phase of breathing.

## 4. Discussion

The present study represents the first report on IOS measurements and ΔX in asthmatic horses in remission of the disease. Horses in remission of severe asthma are of particular interest, because they can be used as a model for subclinical airway obstruction. A previous study, in fact, demonstrated the presence of a residual bronchoconstriction even after one year of treatment with inhaled corticosteroids or strict antigen avoidance [5].

Concerning the measurement technique, IOS generated a spectrum of frequencies ranging from 0.1 to 20 Hz. Nevertheless, it has been demonstrated that the frequencies lower than 10 Hz are the most representative of the lower airways in the equine species [17]. Moreover, in a previous study reporting data obtained in horses by means of forced oscillations (FOT), the frequencies considered as the most sensitive were 1, 2, and 3 Hz [22]. For this reason, we decided to evaluate only the results at 3, 5, and 7 Hz; we excluded the data at lower frequencies because the oscillations generated by the IOS could interfere with higher harmonics of spontaneous respiratory frequencies, and therefore the quality of data could be negatively influenced [23]. For the same reason, and as suggested previously [17], only impedance data showing high values of coherence (>0.85 at 3 Hz and >0.9 at 5 and 7 Hz) were included, in order to optimize the quality of data. Other studies on IOS values in horses with asthma did not report the coherence values [13,14,15,16,18], and therefore the comparison with our data is not possible.

In agreement with previous reports, IOS identified several differences between horses with SEA in exacerbation of the clinical signs and controls [13,14,15,16]. Differences in R were found only at the frequency of 3 Hz for the whole breath and for inspiratory and expiratory R. This result is similar to what reported in a recent study [19]. In human medicine, the increase in R at low frequencies is indicative of the presence of lower airway obstruction during clinical exacerbation of asthma [24]. The absence of differences at higher frequencies is also coherent with previous reports, as SEA horses are characterized by negative frequency dependence of R [16].

In the present study, horses in asthma exacerbation also showed significant lower values of X compared to controls, at all frequencies and for all the phases of breathing. This finding agrees with previous reports [15,19]. Negative values of X at low frequencies reflect peripheral airway obstruction [12]. In humans, X is decreased in the presence of various obstructive respiratory diseases, including asthma, chronic obstructive pulmonary disease (COPD), and emphysema [25].

Moreover, our results showed that the values of ΔX at 3 and 5 Hz in the SEA group in exacerbation were significantly higher than in controls, meaning that the expiratory X was significantly lower than the inspiratory X. Similar findings have been observed in human patients with COPD, but specifically only in patients where the airflow during expiration did not increase despite increasing efforts of the patient. This conditions, called tidal Expiratory Flow Limitation (EFLt), is due to the narrowing of some airways (chock points) consequent from the dynamic compression of peripheral airways [26]. During an IOS measurement, the oscillations cannot penetrate through the choke points and, therefore, impedance data represents the mechanical properties of the part of the lung between choke points and airway opening only. As most of lung compliance is located in the lung periphery (i.e., between chock points and alveoli), when choke points develop, the expiratory reactance drops [20]. In a similar way, exacerbation of SEA is characterized by an early peak of expiration, and a consequent decrease in the expiratory flow [27]. This also may be due to the presence of some choke points that cause a drop in expiratory X and a consequent increase in ΔX. This supports the presence of EFLt in SEA, which may contribute to pulmonary hyperinflation and exercise intolerance, as reported in humans [28].

Finally, IOS allowed to identify significantly lower X7, X3i, X5i, and X7i in SEA horses in remission compared to controls. This is the first report of the sensitivity of IOS in discriminating between healthy and horses in remission of severe asthma. This finding is surprising because negative values of inspiratory reactance are suggestive of restrictive diseases, such as interstitial lung disease, more than obstructive [29]. Moreover, horses with SEA in clinical remission of the clinical signs have normal lung function when evaluated using standard lung mechanics, despite the presence of a residual airway obstruction has been demonstrated [5]. It could be hypothesized that this residual bronchospasm does not interfere with the measurement of expiratory reactance by IOS. Nevertheless, it has been reported that asthmatic horses suffer from a chronic remodeling of the airways, that involves not only the smooth muscle mass [5], but also the vessels [30], the epithelium and the interstitial tissue [31], that is only partially improved by treatment or antigen avoidance. As the presence of pulmonary fibrosis and emphysema induces lower values of inspiratory X in humans [32], it could be speculated that the presence of a persistent subepithelial fibrosis and hyperinflation in horse with SEA in clinical remission [31] could have contributed to the decrease in inspiratory X.

## 5. Conclusions

The within breath analysis of IOS measurement showed some differences between control horses and SEA horses in remission, that could be a promising result for the identification of asthmatic horses in absence of clinical signs. Moreover, the parameter ΔX suggests the presence of EFLt and dynamic airway compression in SEA horses in exacerbation of the clinical signs.

## Figures and Tables

**Table 1:** Results of IOS measurement in the three group. Normally distributed data are displayed as mean ± standard deviation. Non-normal distributed values are presented as median and interquartile range. (* = *p* < 0.05; ** = *p* < 0.01; *** = *p* < 0.001).

	Controls (cmH_2_O * s/L)	Remission (cmH_2_O * s/L)	Exacerbation (cmH_2_O * s/L)
**Mean**			
R3	0.68 (±0.21)	0.87 (±0.35)	0.142 (±0.24) ***
R5	0.75 (±0.22)	0.86 (±0.32)	0.93 (±0.09)
R7	0.91 (±0.24)	0.92 (±0.34)	0.82 (±0.12)
X3	0.12 (±0.06)	−0.03 (±0.14)	−0.97 (±0.56) ***
X5	0.21 (±0.06)	−0.01 (±0.2)	−0.67 (±0.35) ***
X7	0.24 (±0.11)	−0.03 (±0.2) *	−0.49 (±0.24) ***
**Inspiratory**			
R3i	0.60(0.053–0.77)	0.95 (0.76–1.05)	1.2 (1.17–1.35) **
R5i	0.72 (±0.2)	0.88 (±0.32)	0.92 (±0.07)
R7i	0.87 (±0.21)	0.93 (±0.31)	0.80 (±0.19)
X3i	0.16 (±0.09)	−0.06 (±0.15) **	−0.39 (±0.14) ***
X5i	0.22 (±0.05)	−0.05 (±0.17) **	−0.31 (±0.14) ***
X7i	0.28 (±0.01)	−0.05 (±0.18) *	−0.18 (±0.28) ***
**Expiratory**			
R3e	0.71 (±0.24)	0.85 (±0.35)	1.4 (±0.27) ***
R5e	0.79 (±0.24)	0.85 (±0.31)	0.9 (±0.11)
R7e	0.97 (±0.27)	0.92 (±0.35)	0.8 (±0.14)
X3e	0.08 (±0.05)	−0.02 (±0.15)	−1.15 (±0.68) ***
X5e	0.19 (±0.08)	0.01 (±0.21)	−0.8 (±0.43) ***
X7e	0.2 (±0.13)	−0.02 (±0.22)	−0.54 (±0.27) ***
**Delta (Δ) X**			
ΔX3	0.08 (±0.07)	−0.05 (±0.1)	0.76 (±0.58) **
ΔX5	0.03 (±0.04)	−0.06 (±0.1)	0.47 (±0.32) **
ΔX7	0.05 (0.03–0.07)	−0.01 (−0.14–0.04)	0.16 (0.13–0.47)

## Data Availability

The data presented in this study are available on request from the corresponding author.
